# Kinetics of cancer: a method to test hypotheses of genetic causation

**DOI:** 10.1186/1471-2407-5-163

**Published:** 2005-12-21

**Authors:** Steven A Frank, Peng-Chieh Chen, Steven M Lipkin

**Affiliations:** 1 Department of Ecology and Evolutionary Biology, University of California, Irvine, CA 92697-2525, USA; 2 Divisions of Hematology-Oncology and Epidemiology, Departments of Medicine and Biological Chemistry, University of California, Irvine, CA 92697-4038, USA

## Abstract

**Background:**

Mouse studies have recently compared the age-onset patterns of cancer between different genotypes. Genes associated with earlier onset are tentatively assigned a causal role in carcinogenesis. These standard analyses ignore the great amount of information about kinetics contained in age-onset curves. We present a method for analyzing kinetics that measures quantitatively the causal role of candidate genes in cancer progression. We use our method to demonstrate a clear association between somatic mutation rates of different DNA mismatch repair (MMR) genotypes and the kinetics of cancer progression.

**Methods:**

Most experimental studies report age-onset curves as the fraction diagnosed with tumors at each age for each group. We use such data to estimate smoothed survival curves, then measure incidence rates at each age by the slope of the fitted curve divided by the fraction of mice that remain undiagnosed for tumors at that age. With the estimated incidence curves, we compare between different genotypes the median age of cancer onset and the acceleration of cancer, which is the rate of increase in incidence with age.

**Results:**

The direction of change in somatic mutation rate between MMR genotypes predicts the direction of change in the acceleration of cancer onset in all 7 cases (*p *˜ 0.008), with the same result for the association between mutation rate and the median age of onset.

**Conclusion:**

Many animal experiments compare qualitatively the onset curves for different genotypes. If such experiments were designed to analyze kinetics, the research could move to the next stage in which the mechanistic consequences of particular genetic pathways are related to the dynamics of carcinogenesis. The data we analyzed here were not collected to test mechanistic and quantitative hypotheses about kinetics. Even so, a simple reanalysis revealed significant insights about how DNA repair genotypes affect separately the age of onset and the acceleration of cancer. Our method of comparing genotypes provides good statistical tests even with small samples for each genotype.

## Background

Experimental studies of cancer genetics often compare a control population of mice to an experimental population in which a candidate cancer gene has been altered. The experiment measures the age of tumor onset in particular tissues for the control and experimental groups.

If the age-onset curve for the experimental group is shifted to an earlier age relative to the control group, then the altered gene plays a role in the rate of cancer progression. The typical analysis often stops at a qualitative conclusion: either the gene does or does not play a role.

We present a method to extract quantitative information from such studies – information about the kinetics of progression. For example, does the altered gene simply shift onset to an earlier age or does it increase the rate at which incidence increases with age? Among a set of altered genotypes, how does abrogating different functions affect the age of onset and the rate of increase in incidence?

The way to measure causality of genes in cancer is to measure the quantitative effects of genes on the age-onset patterns of disease. Thus, methods to extract and analyze kinetic information from experimental studies provide an important step in the development of the subject.

Here, we develop the methods for quantitative analysis and apply those methods to experimental data of age-onset patterns for different DNA mismatch repair (MMR) genotypes. The MMR genotypes have different rates of somatic mutation, so our kinetic analysis provides information about how somatic mutation rates affect quantitative aspects of cancer progression.

## Methods

### Data collection

We analyze data originally described by Chen et al. [1]. We supplemented the data with additional observations collected by the same methods and obtained following publication of [1]. When a mouse became morbid, it was sacrificed and surveyed; all malignancies were confirmed by histopathology. Each mouse was scored as positive or negative for lymphoma, GI adenoma, or GI carcinoma. A single mouse could be positive for both lymphoma and a GI tumor; however, GI adenomas and carcinomas are mutually exclusive.

Over all 135 mice in our data, 91 were diagnosed with lymphoma. Consistent with other studies of MMR deficient mice, the great majority of GI tumors were adenomas: 62 had GI adenomas and 7 had GI carcinomas. Below, we discuss the problem of analyzing the different progression stages represented by GI adenomas and carcinomas. Of the 135 mice, 6 died of unknown cause and so were scored negatively for both lymphomas and GI cancers. We include those 6 mice in our analyses because their ages of death contribute to the changing base population of mice at risk at different ages.

By using observed morbidity to measure age of onset, there is a time lag between the final molecular progression event that leads to tumor growth and the observation of morbidity. We tested how time lags affected our quantitative methods by reanalyzing our data under the assumption that the age of onset is one or two months earlier than the recorded age of morbidity. Time shifts had only small quantitative effects on our estimates of kinetics, and the lags did not affect any comparisons or statistical evaluations based on the aggregate of the various comparisons (results for time lag adjustments not shown). The insensitivity of our results to the magnitude of time lags arises from the fact that we measure kinetics relative to logarithmic scaling of time, and potential lags cause only small distortions along the time dimension.

See Additional file 1: figure1Data.xls for the data analyzed in this paper.

### Quantitative analysis

**Figure 1:**
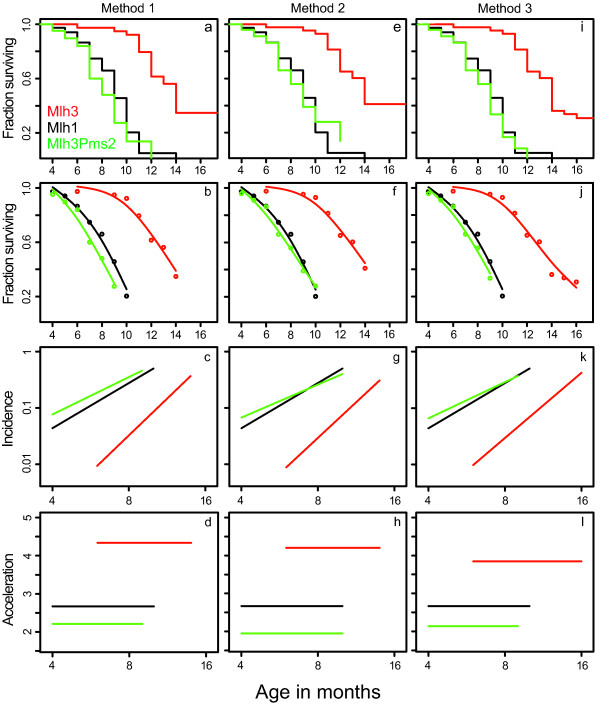
**GI tumor kinetics for three MMR knockout mouse genotypes**. For each genotype, both alleles at each locus were knocked out. **a **Kaplan-Meier estimate [2] at each age of the fraction of mice that have not yet developed GI tumors among the population of mice that remain at risk. **b **Smoothed curve fit to the estimated survival curve in **a **using the smooth.spline function of the R computing language [2] with the smoothing parameter set to 0.5. **c **Incidence of GI tumors on log-log scales. **d **The acceleration of tumor onset calculated from the slope of the lines in **c**. Each column of plots corresponds to one of the three methods for dealing with the 7 observed GI carcinomas; we explained the different methods in the text. In our calculations, we truncated the curves at survival fractions below 0.2 because the errors in incidence estimates rise rapidly at low levels of survival. The spreadsheet included in the supplemental materials provides a full listing of the data used to generate Figures 1 and 2.

**Figure 2:**
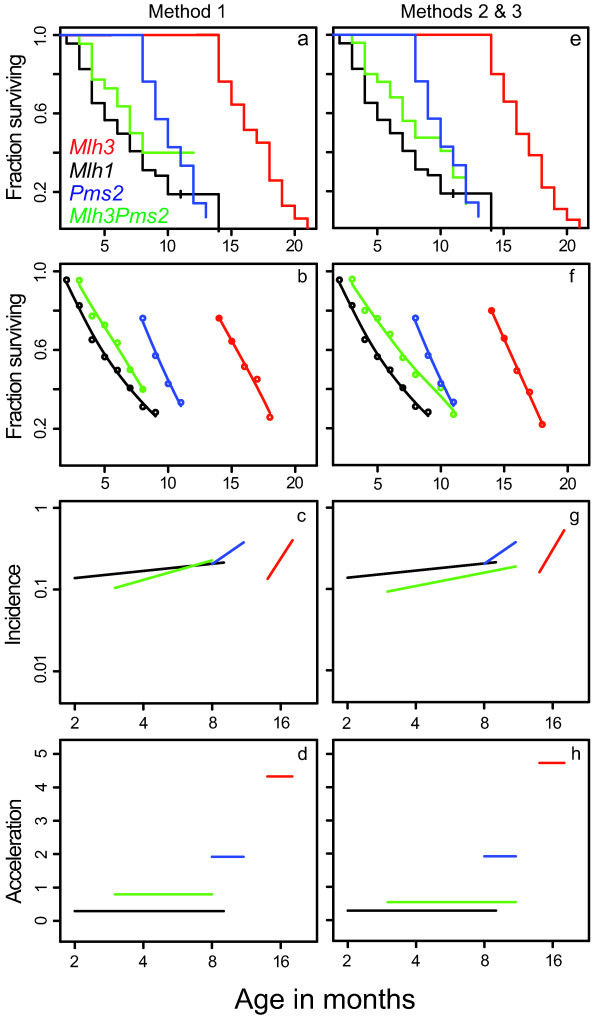
**Lymphoma kinetics for four MMR knockout mouse genotypes**. Lymphoma data plotted with the same style as the plots in Figure 1. The columns show the different methods for dealing with the 7 mice who had GI carcinomas, as explained in the text. Methods 2 and 3 are equivalent for analysis of the lymphoma data.

Figure 1a shows age-onset data for gastrointestinal (GI) tumors for different MMR genotypes. This plot presents the Kaplan-Meier estimate [2] at each age of the fraction of mice that have not yet developed GI tumors among the population of mice that remain at risk. A mouse diagnosed with lymphoma but no GI tumor is scored as removed from the population at the age of diagnosis. This type of Kaplan-Meier curve is often referred to as a survival curve, *S(t)*, because it provides an estimate of the expected fraction of mice that would be diagnosed with GI cancer at each age, *t*, if GI cancer were the only cause of morbidity. We used an analogous method for the lymphoma data in Figure 2a, treating those mice with GI tumors but no lymphoma as removed from the population at the age of diagnosis.

Figure 1b shows a smoothed survival curve fit to the Kaplan-Meier plot. From the survival curve, *S(t)*, in Figure 1b, we estimate for each genotype the incidence rate, or probability of GI cancer per month at age *t*, as *I(t) *= -(d*S(t)*/d*t*)/*S(t)*, which is proportional to the estimated number of new cases per unit time divided by the number of undiagnosed individuals at that time.

The classical way of quantitatively evaluating incidence data comes from the multistage theory of carcinogenesis [3]. According to that theory, if there are *n *stages in the development of cancer, and the transition rate between stages is *u*, then *I(t) ≈ u^*n*^t^*n-1*^/n-1!*, where *u *is often related to the rate of somatic mutation. When testing specific mechanistic hypotheses, one can use other forms of the incidence function that account for additional processes, such as clonal expansion of precancerous lesions [4].

It is useful to analyze the logarithm of incidence versus the logarithm of age. In the classical form, taking the logarithm of both sides yields log(*I(t)*) ˜ log(*u^*n*^/n-1!*) + (*n-1*)log(*t*). Similar expressions would be obtained by using other theoretical predictions for incidence.

Our analysis does not depend on any particular mathematical theory of progression and incidence. Instead, we formulate comparative hypotheses about the expected direction of change in cancer progression kinetics when comparing different genotypes. Our simple qualitative hypotheses do not depend on detailed assumptions about progression dynamics.

## Results

Figure 1c shows plots of log(*I(t)*) versus log(*t*), where we used the smoothed curves in Figure 1b to calculate *I(t)*, and then fit straight lines through the values of *I(t) *on a log-log scale. We fit straight lines because the data provide enough information to get a good estimate of the slope, but not enough information to provide a good estimate of the curvature of the log-log plots at different ages.

The incidence, *I(t)*, is the rate of cancer at each age. It is often useful to study how cancer incidence changes with age. Changes in the incidence rate can be measured by the acceleration, which is the derivative (slope) of the incidence (rate) curve; acceleration is simply a measure of how fast the incidence rate, *I(t)*, changes with age. We measure the log-log acceleration of cancer with age, which is dlog(*I(t)*)/dlog(*t*) [5,6]. Figure 1d shows the log-log accelerations for each genotype.

To estimate survival curves, we had to decide how to treat the seven mice with GI carcinomas (see Methods and the full data set in the supplemental information). We used three alternative methods: (1) delete the seven mice from all analyses for both GI cancers and lymphoma; (2) for GI cancers, treat the GI carcinomas as a different disease and score those mice as removed from the population at the age of diagnosis, and score lymphomas as present or absent in the normal way for those seven mice; and (3) score the GI carcinomas as adenomas but set the age of onset one month earlier than observed because carcinomas represent a later stage in progression than do adenomas, and score lymphomas as present or absent in the normal way for those seven mice.

Figure 1 shows our analysis of GI data for the three different methods of treating GI carcinomas. Figure 2 shows our analysis of lymphoma data for the three methods: note that methods 2 and 3 are equivalent for lymphomas. The qualitative patterns remain unchanged between methods, and the quantitative changes are small.

## Discussion

Multistage theory for the kinetics of carcinogenesis makes three qualitative predictions. First, the fewer the number of steps in progression that must be passed, the lower the acceleration of cancer with age. In mouse experiments, the theory predicts that abrogation of tumor suppressor functions or introduction of oncogenes reduces the acceleration. Second, small to moderate increases in the mutation rate, *u*, cause greater cancer incidence at earlier ages but do not affect the acceleration. Third, large increases in *u *can cause such rapid transitions between stages that certain mutations required for carcinogenesis may no longer limit the rate of tumor formation. If some transitions no longer limit the kinetics of carcinogenesis, the number of rate-limiting steps decreases, and the acceleration declines.

MMR genotypes affect both mutation rate and apoptosis in response to DNA damage. Apoptosis has tumor suppressor function and may often be a rate-limiting step in carcinogenesis. Previous work [1,7] showed that the mutation rates for the four knockout genotypes can be ordered as *Mlh3 < Pms2 < Mlh1 ˜ Mlh3Pms2*, and the decreased apoptosis in response to DNA damage of the four genotypes can be ordered as *Mlh3 ˜ Pms2 < Mlh1 ˜ Mlh3Pms2.*

Table 1 shows that differences in mutation rate predict the direction of change in acceleration and median age of onset. Note that it is possible to have later age of onset and lower acceleration, so acceleration and age of onset are two independent dimensions of the kinetics. The direction of change in mutation rate predicts the direction of change in the acceleration in all 7 cases (*p *˜ 0.008), with the same result for the association between mutation rate and age of onset. Differences in anti-apoptotic effects (not shown) also predict the direction of change in acceleration and age of onset.

## Conclusion

We presented a method to test causal hypotheses about cancer progression kinetics. Our method is based on the concept that a genotype has a causal influence on carcinogenesis only to the extent that the genotype affects the age-specific incidence curves of cancer.

Limited sample sizes present the greatest problem in studies that estimate age-specific incidence for particular genotypes. To get around this limitation, we formulated our hypotheses as predictions about the direction of change in comparisons between genotypes. For example, we predicted that acceleration would decline in a sample with relatively stronger defects in mismatch repair when compared against a sample with relatively weaker defects in mismatch repair.

By formulating each key prediction in a comparative way, mouse data with small sample sizes can be used. Each comparison provides a single binary outcome that represents either a success or failure of the theory to predict the direction of change in some attribute of incidence kinetics. The binary outcomes can be aggregated to form a nonparametric test based on the binomial distribution. This allows our approach to be applied to small samples of mice in each genotype. The effective sample size comes from the number of comparisons.

Over the past few years, vast resources have been expended on animal experiments that compare survival curves for different genotypes. If these sorts of experiments were designed and analyzed with kinetics in mind, the research could move to the next stage in which the mechanistic consequences of particular genetic pathways are related to the dynamics of carcinogenesis. The data we analyzed here were not collected to test mechanistic and quantitative hypotheses about kinetics. A simple reanalysis revealed significant insights about how DNA repair genotypes affect separately the age of onset and the acceleration of cancer.

## Competing interests

The author(s) declare that they have no competing interests.

## Authors' contributions

SAF developed the theory and data analysis and wrote the first draft. PCC did the experiments and provided the data for analysis. SML conceived the laboratory part of the study and helped to draft the manuscript. All authors read and approved the final manuscript.

## Pre-publication history

The pre-publication history for this paper can be accessed here:



## Supplementary Material

Additional File 1 The file figure1Data.xls contains the data we used for our analyses. This file is an Excel spreadsheet. Click here for file

## Figures and Tables

**Table 1:** Comparison of tumor kinetics for four different MMR genotypes. The '+' and '-' symbols show the direction of change for each comparison. In each comparison, the genotype with the higher mutation rate had a lower acceleration and median age of onset.

Comparison	Type	Acceleration	Age	Mutation
*Mlh3 *v *Mlh3Pms2*	GI tumor	+	+	-
*Mlh3 *v *Mlh1*	GI tumor	+	+	-
*Mlh3 *v *Mlh3Pms2*	Lymphoma	+	+	-
*Mlh3 *v *Mlh1*	Lymphoma	+	+	-
*Pms2 *v *Mlh3Pms2*	Lymphoma	+	+	-
*Pms2 *v *Mlh1*	Lymphoma	+	+	-
*Mlh3 *v *Pms2*	Lymphoma	+	+	-
